# Influence of an InGaN superlattice pre-layer on the performance of semi-polar (11–22) green LEDs grown on silicon

**DOI:** 10.1038/s41598-020-69609-4

**Published:** 2020-07-28

**Authors:** X. Zhao, K. Huang, J. Bruckbauer, S. Shen, C. Zhu, P. Fletcher, P. Feng, Y. Cai, J. Bai, C. Trager-Cowan, R. W. Martin, T. Wang

**Affiliations:** 1 Department of Electronic and Electrical Engineering, University of Sheffield, Sheffield, S1 3JD UK; 2 Department of Physics, SUPA,, University of Strathclyde, Glasgow, G4 0NG UK

**Keywords:** Materials science, Optics and photonics

## Abstract

It is well-known that it is crucial to insert either a single InGaN underlayer or an InGaN superlattice (SLS) structure (both with low InN content) as a pre-layer prior to the growth of InGaN/GaN multiple quantum wells (MQWs) served as an active region for a light-emitting diode (LED). So far, this growth scheme has achieved a great success in the growth of III-nitride LEDs on *c-plane* substrates, but has not yet been applied in the growth of any other orientated III-nitride LEDs. In this paper, we have applied this growth scheme in the growth of semi-polar (11–22) green LEDs, and have investigated the impact of the SLS pre-layer on the optical performance of semi-polar (11–22) green LEDs grown on patterned (113) silicon substrates. Our results demonstrate that the semi-polar LEDs with the SLS pre-layer exhibit an improvement in both internal quantum efficiency and light output, which is similar to their *c-plane* counterparts. However, the performance improvement is not so significant as in the *c-plane* case. This is because the SLS pre-layer also introduces extra misfit dislocations for the semi-polar, but not the *c-plane* case, which act as non-radiative recombination centres.

## Introduction

The last two decades have seen an unprecedented success in developing III-nitride optoelectronics on foreign substrates, such as sapphire, which exhibit large lattice-mismatches with GaN. This has led to great success in commercialising III-nitride based emitters (light emitting diodes (LEDs) and laser diodes (LDs)), in particular blue LEDs. However, it is worth highlighting that a number of fundamental issues are still yet to be fully understood. Dislocations have been proven as non-radiative recombination centres (NRCs)^1^^,^ and thus considerable efforts have been devoted to the development of high quality GaN on widely used sapphire or silicon substrates. By contrast, III-nitride LDs with reasonably good performance and long life-time can only be obtained by homo-epitaxial growth on GaN substrates or templates.

It is also well-known that it is crucial to insert a single InGaN underlayer or an InGaN/GaN superlattice structure (SLS) as a pre-layer prior to the growth of InGaN/GaN multiple quantum wells (MQWs) as an emitting region in an LED. The performance of the III-nitride LEDs are then dramatically improved^2–10^, although the pre-layer is typically grown at a low temperature which generates extra defects^8–10^. Furthermore, even for the growth on GaN substrates (where the dislocation density is significantly low), III-nitride LEDs without any pre-layer exhibit much worse performance than those with a pre-layer but grown on sapphire^8–10^. It suggests that factors in addition to dislocations also play a vital role in determining the performance of III-nitride LEDs.

In order to address this issue, a number of models have been proposed to provide a mechanism for the enhanced performance of III-nitride LEDs grown with a pre-layer^2–10^. So far, the most convincing model identifies point defects induced by vacancies as an important additional source of NRCs^8–10^. Furthermore, it has been demonstrated that the density of point defects in InGaN/GaN MQWs in the active region of an LED is significantly reduced if a pre-layer is prepared prior to the growth of the active region^9^. Comparative experiments based on LEDs grown on sapphire and GaN substrates with or without an InGaN underlayer as a pre-layer provide solid evidence to support this model^9^. The enhanced LED performance is due to a significant reduction in the density of point defects as a result of the insertion of such a pre-layer grown at a low temperature, while the experiment has also ruled out any other possibilities, such as enhanced performance due to a change in surface morphology^4^ or in strain state^7^ or a reduction in internal electric fields^6^. However, so far, all the research on this issue is restricted to III-nitride LEDs on *c-plane* substrates.

Very recently, semi-polar III-nitride LEDs, in particular III-nitride LEDs grown on the (11–22) semi-polar GaN surface, have drawn increasing attention due to a growing demand for developing LEDs emitting at longer wavelengths, such as green, yellow or even red^11,12^. Semi-polar LEDs exhibit intrinsic advantages compared with their *c-plane* counterparts, such as significant reduction in polarization^12,13^ and enhanced InN incorporation efficiency in InGaN^14,15^, both of which are critically important for developing long wavelength LEDs. So far, impressive results on semi-polar LEDs have been obtained on extremely expensive and size limited GaN substrates^16,17^. Although the performance of semi-polar LEDs is still far from that of blue LEDs on *c-plane* substrate, it has also been recognized that the major advantage of semi-polar LEDs, in particular semi-polar (11–22) LEDs, is due to the development of LEDs emitting at longer wavelengths, such as yellow^11,12^.

A careful literature review shows that all semi-polar LEDs reported so far do not utilize growth schemes involving either an InGaN underlayer or InGaN SLS as a pre-layer. Therefore, it is worth investigating whether the pre-layer plays a similar role in semi-polar LED to their *c-plane* counterparts. Of course, it is more attractive to investigate the issue on semi-polar LEDs grown on industry-compatible substrates, such as sapphire or silicon, in particular the latter due to an increasing demand for developing an integration of III-nitride optoelectronics and silicon technologies.

Prior to starting the investigation, the crystal quality of semi-polar (11–22) GaN on silicon needs to be improved to a point where the crystal quality of the (11–22) GaN is similar to or at least approach its *c-plane* counterpart on silicon. In order to address the material issues, our group spent the last decade on establishing a number of cost-effective approaches to achieve semi-polar (11–22) GaN with high quality on either *m-plane* sapphire or patterned (113) silicon substrates^11,12,18–20^. For the latter, the combination of a stripe-patterning process and anisotropic chemical etching is used to form parallel grooves with an optimised depth on (113) silicon, on which selective growth on the sidewall {111} facet of the silicon is performed. As a result, (11–22) GaN with significantly improved crystal quality on silicon has been achieved, and InGaN green LEDs on such a semi-polar GaN template have been demonstrated^20^. These provide us with a timely opportunity to investigate the influence of an InGaN pre-layer on the performance of semi-polar LEDs grown on silicon, and then identify whether the InGaN pre-layer plays a similar role in semi-polar LEDs to their *c-plane* counterparts.

In this study, two semi-polar (11–22) green LEDs with a similar structure have been grown on patterned (113) silicon substrates: one with 15 pairs of InGaN/GaN SLS as a pre-layer and another without any pre-layer. Detailed optical investigations demonstrate that an enhanced internal quantum efficiency (IQE) and a reduction in efficiency droop have been obtained on the semi-polar LEDs with the SLS pre-layer compared with the one without any pre-layer. This is similar to the observations on *c-plane* counterparts as expected. However, it is notable that the improvement is not so significant as those for *c-plane* LEDs. Cathodoluminescence experiments identify that the reason is likely related to the generation of extra misfit dislocations by the insertion of the SLS pre-layer although the InN content in the InGaN of the SLS is less than 3% and thus the lattice-mismatch in the SLS is quite small. Therefore, the insertion of the SLS pre-layer leads to a reduction in point defect density, improving the IQE. However, these misfit dislocations act as NRCs and reduce the IQE. Consequently, the overall effect is that the semi-polar green LED with the SLS pre-layer exhibits an improved IQE, but the improvement is not so significant as observed for its *c-plane* counterparts.

## Results and discussion

**Figure 1:**
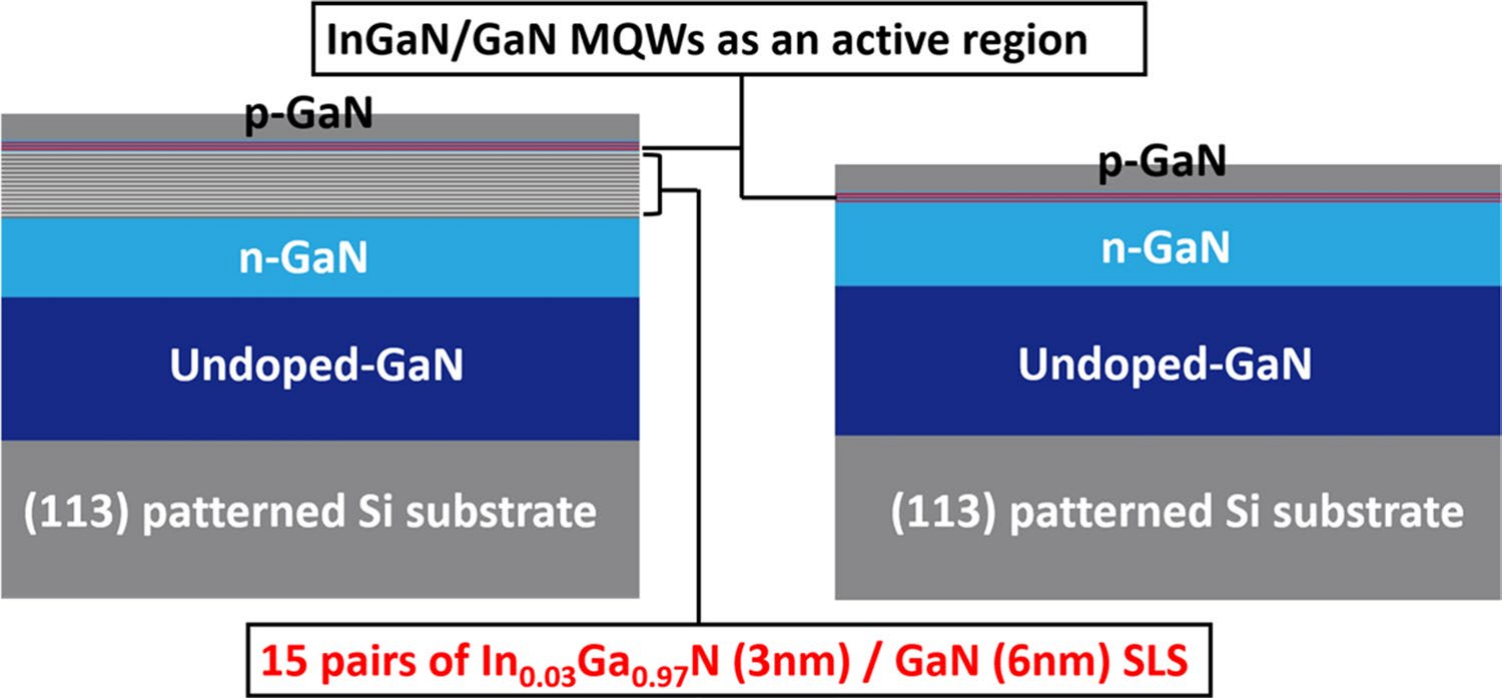
Schematic of the semi-polar (11–22) LEDs with and without the SLS pre-layer grown on (113) patterned Si substrates.

Two semi-polar (11–22) green LEDs with a similar structure have been grown on our semi-polar (11–22) GaN templates with high crystal quality obtained on patterned (113) Si substrates. For the detail of the patterned (113) silicon and the material characterization of the semi-polar (11–22) GaN templates, please refer to our recently published paper^20^. The LED structure with a SLS pre-layer has a 1.2 µm n-type GaN layer and then 15 pairs of In_0.03_Ga_0.97_ N/GaN SLS (3 nm/6 nm), followed by a threefold InGaN/GaN MQW as an active region, with 3 nm quantum wells and 9 nm barriers (the InN content in the InGaN quantum well is estimated to be 25%) and a final 150 nm p-type GaN layer, while the LED without any pre-layer has an identical structure except for the 15 pairs of SLS. Figure 1 schematically illustrates these two LED structures with and without the SLS pre-layer.

**Figure 2:**
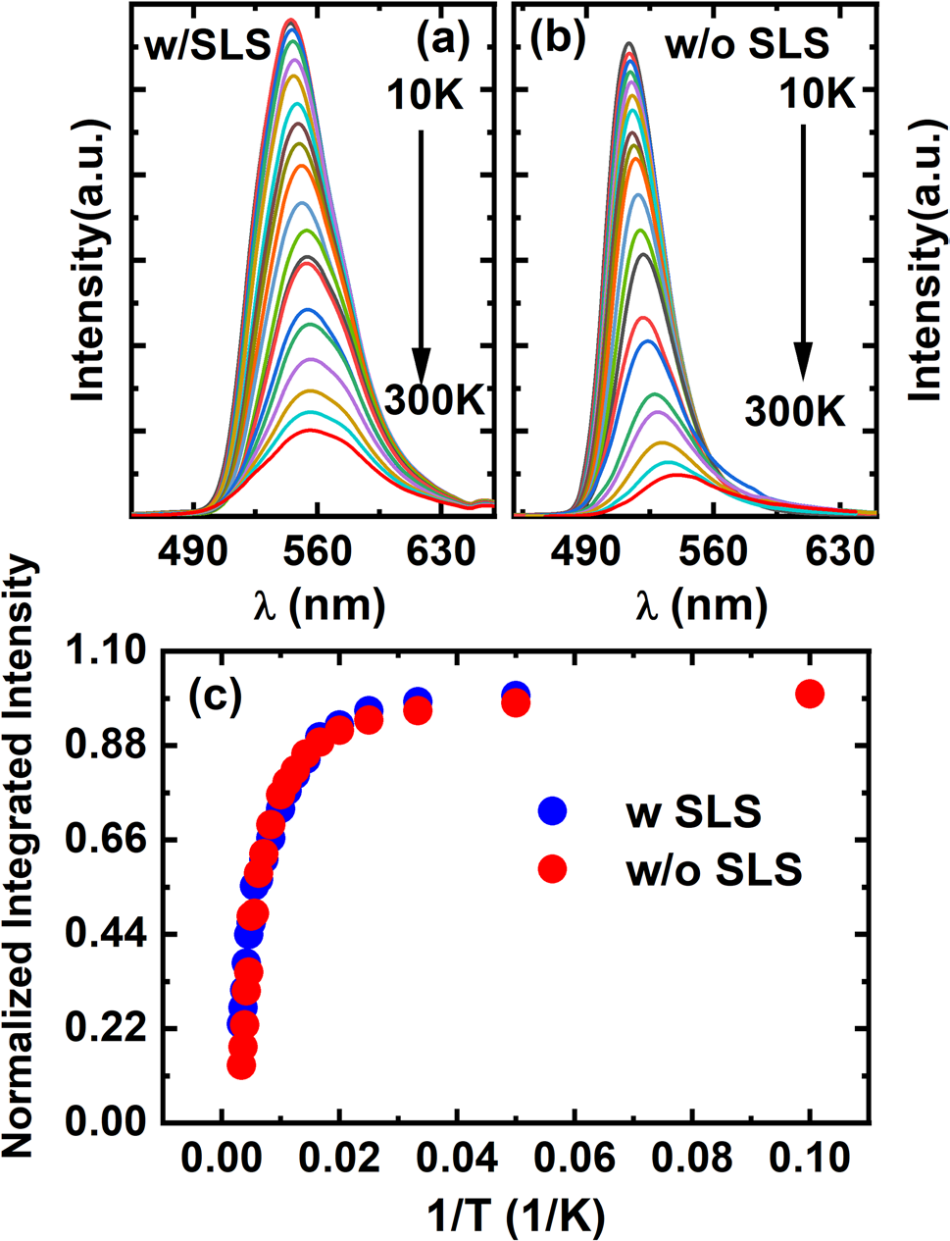
Temperature dependent PL spectra of the semi-polar LEDs with **(a)** and without **(b)** the SLS pre-layer; Integrated PL intensity of the the semi-polar (11–22) LEDs with and without the SLS pre-layer as a function of temperature **(c)**.

Figure 2a and b show the photoluminescence (PL) spectra of the semi-polar LED samples with and without the SLS pre-layer as a function of temperature ranging from 10 to 300 K. Both samples show a strong emission peak at around 550 nm. Figure 2c shows the normalized integrated PL intensities of the two samples as a function of temperature, from which the room temperature IQE can be estimated. This is a standard, widely used method, which assumes 100% IQE at low temperature. The sample with the SLS pre-layer exhibits a 23% IQE at room temperature, which is considerably higher than the 13% IQE of the sample without a pre-layer. This demonstrates that the insertion of the SLS pre-layer indeed enhances the IQE of the semi-polar LED by a factor of 1.8. However, compared to c-plane samples with and without a pre-layer, this enhancement is significantly lower. For example, it has been reported that the quantum efficiency can be enhanced by a factor of 3.5 for *c-plane* LEDs featuring a SLS pre-layer^9^.

**Figure 3:**
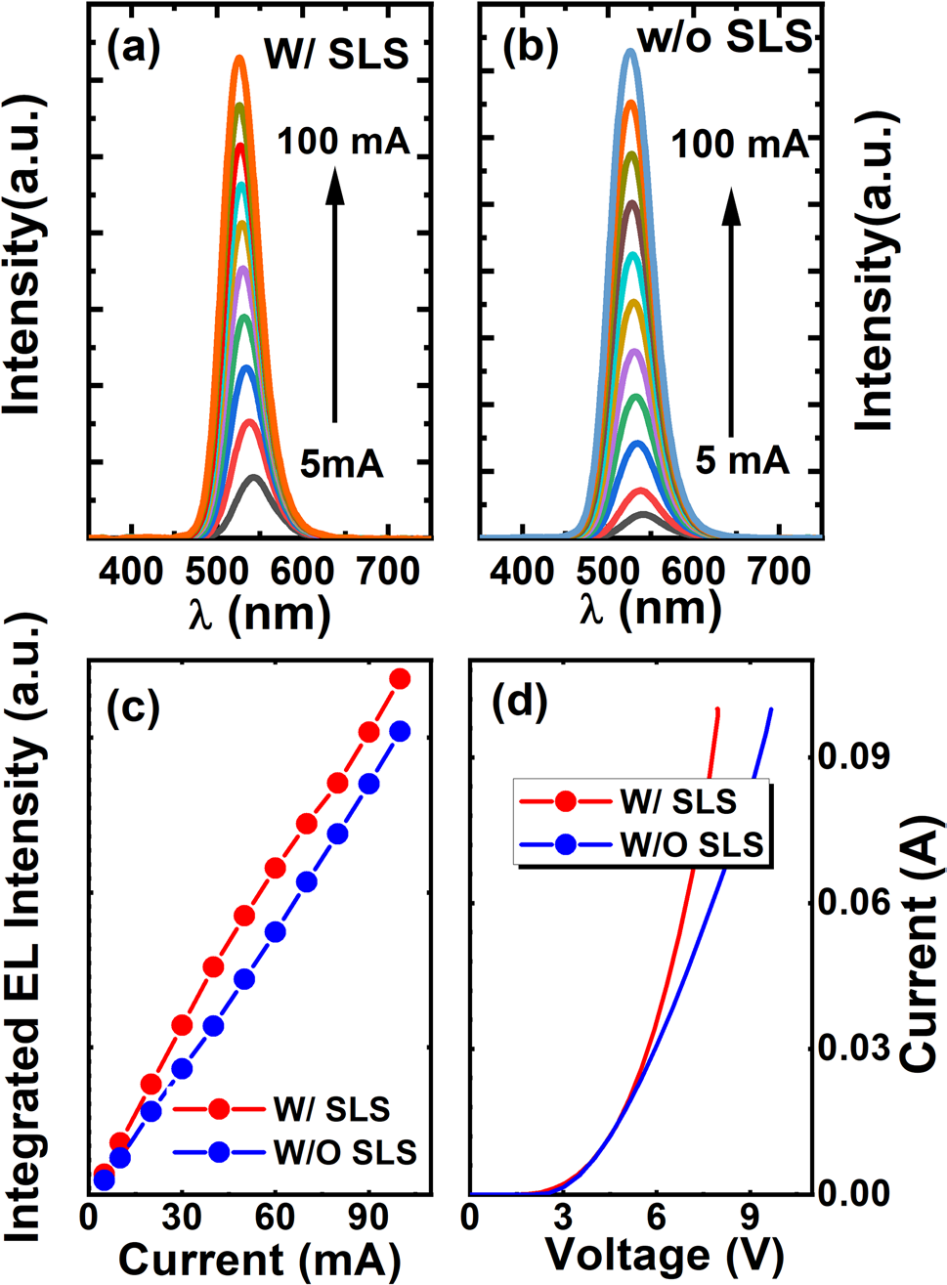
Electroluminescence spectra of the semi-polar (11–22) LEDs with **(a)** and without **(b)** the SLS pre-layer as a function of injection current; light output powers (i.e., integrated EL spectra) of the two semi-polar LEDs as a function of injection current **(c)**; and I–V characteristic of the two semi-polar LEDs **(d)**.

Figure 3a and b show the EL spectra of these two LEDs (with and without the SLS pre-layer) in a bare-chip form (i.e., without any packaging) as a function of injection current measured under identical conditions. In both cases, the EL intensity increases with increasing injection current. Figure 3c provides more details of the light output (i.e., integrated EL intensity) of both LEDs as a function of injection current, clearly demonstrating that the light output of the LED with the SLS pre-layer is higher than that of the LED without a pre-layer. These results are in a good agreement with the IQE measurements as shown in Fig. 2. For example, the light output of the LED with the SLS pre-layer is enhanced by 30% compared with the LED without a pre-layer at a 20 mA injection current. This further proves the enhancement in light output as a result of utilizing a SLS pre-layer. Figure 3d shows the current–voltage characteristics of the samples with and without the SLS pre-layer, which are fairly similar. This indicates that the insertion of the SLS pre-layer has not significantly affected the electrical properties.

**Figure 4:**
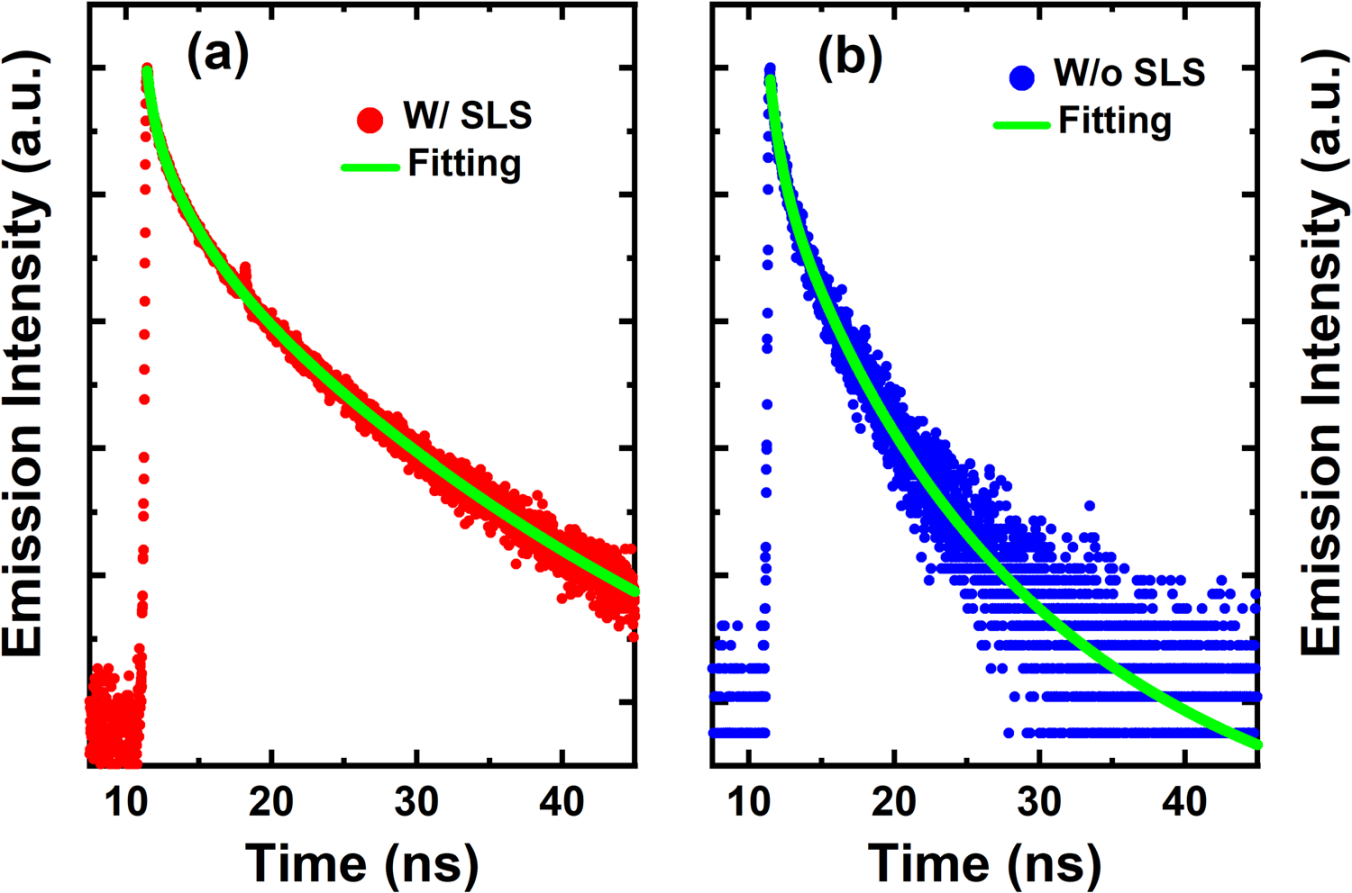
Time-resolved PL spectra of the semi-polar LEDs with (**a**) and without (**b**) the SLS pre-layer, measured at a low temperature.

To further investigate the influence of the SLS pre-layer on the performance of semi-polar LEDs, time resolved PL (TRPL) measurements have been conducted on both samples at a low temperature (10 K). Figure 4a and b show the TRPL traces of the two samples measured under identical conditions at 10 K. A standard bi-exponential model is used, and thus TRPL traces *I(t)* can be described by^21–23^

1 $$I(t) = A_{1} \exp ( - t/\tau_{1} ) + A_{2} \exp ( - t/\tau {}_{2})$$

where the 1st and 2nd items represent the fast and slow components, respectively; A_1_ and A_2_ are constants; and τ_1_ and τ_2_ are the decay lifetimes of the two exponential components.

The fitting results have been plotted as dashed lines. From the bi-exponential fitting, the decay rates for the fast component and the slow component of the sample with the SLS pre-layer have been obtained as 0.91 ns and 10.53 ns, respectively, while the sample without a pre-layer shows a fast decay rate of 0.89 ns and a slow decay component of 7.56 ns, respectively. It can be seen that the fast decay rates for the two samples are very similar, indicating that the inserted SLS pre-layer does not change the polarization-induced QCSE as the polarization in the InGaN/GaN MQWs in both (11–22) semi-polar LEDs is relatively weak due to the nature of semi-polar LEDs^11^. However, the sample without a pre-layer exhibits a considerably faster decay rate for the slow component than the sample with the SLS pre-layer, implying the re-absorption of the emission from the SLS pre-layer whose emission is centred at 383 nm. The emission from the SLS pre-layer will be discussed below.

In order to investigate the impact of the insertion of the SLS pre-layer on the spatial dependence of the luminescence properties, cathodoluminescence (CL) measurements have been performed at room temperature. This provides a further insight on defect recombination on a micro/nano meter length scale^24^.

**Figure 5:**
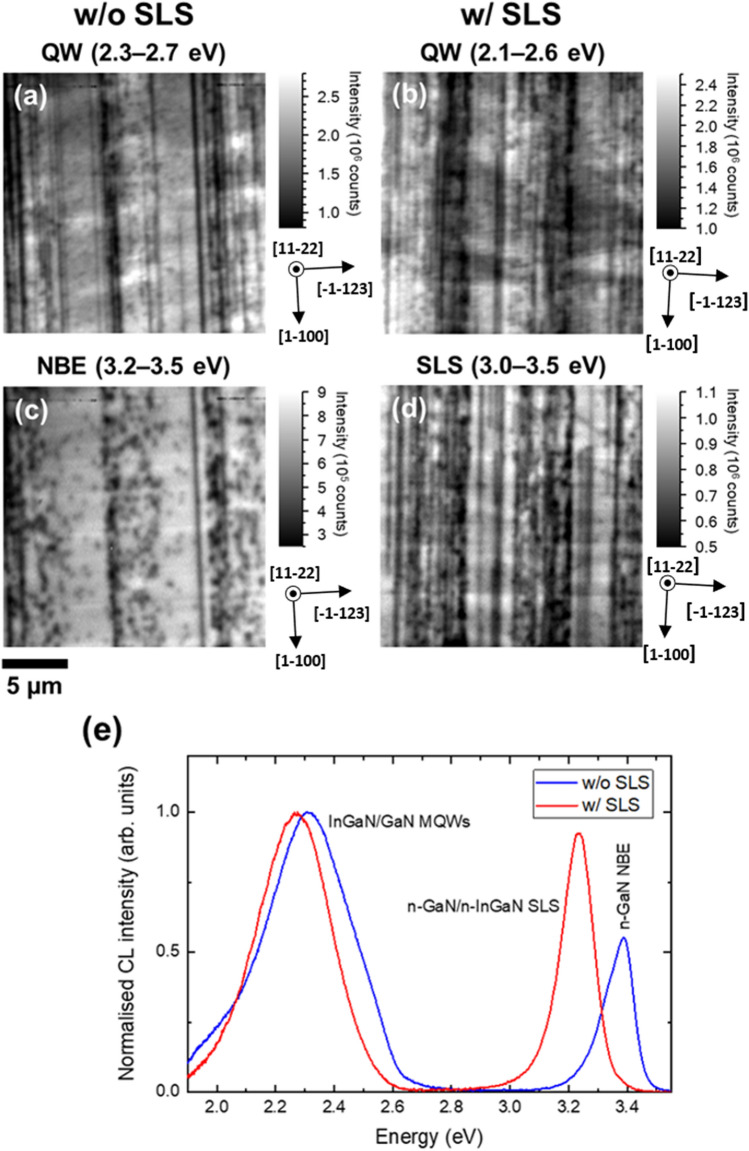
Plan-view CL imaging at room temperature: Integrated CL intensity images of the InGaN/GaN MQW peak of the LEDs **(a)** without and **(b)** with the SLS pre-layer. **(c)** Integrated CL intensity image of the GaN NBE peak of the LED without the pre-layer. **(d)** Integrated CL intensity image of the InGaN/GaN SLS peak of the LED with the pre-layer. **(e)** Mean CL spectra of the LEDs with and without SLS pre-layer.

Figure 5a,c show plan-view integrated CL intensity images of the InGaN/GaN MQW peak and the near band edge (NBE) emission peak of the sample without a pre-layer, respectively. Figure 5b,d display the integrated CL intensity image of the InGaN/GaN MQW peak and the InGaN/GaN SLS emission peak of the sample with the SLS pre-layer, respectively. Both are measured using an electron beam energy of 5 keV. In the case of the sample without the pre-layer the electron beam penetrated the MQW structure and also excited the n-doped GaN underneath. Whereas for the sample with the pre-layer, the electron beam did not reach the GaN, only penetrating into the SLS pre-layer, and only the emission from the InGaN/GaN SLS is observed in addition to the MQW emission for this sample. Figure 5e shows normalized mean CL spectra of both samples. The emission peak from the InGaN/GaN SLS is centred around 3.23 eV (383 nm) compared with the GaN NBE peak at 3.39 eV (366 nm) due to the low InN content InGaN layer (around 3%) in the SLS. The MQW peak also appears at a slightly different position, which is most likely due to the presence of the SLS pre-layer having an effect on the growth of the subsequent MQW structure.

In all four intensity images in Figs. 5a–d, a number of dark lines and dark spots can be clearly observed. As for *c-plane* material, dark spots are associated with non-radiative recombination at threading dislocations. Dark lines in CL images of semi-polar III-nitrides are generally caused by non-radiative recombination at stacking faults or misfit dislocations^25,26^. The density of dark lines is higher in Fig. 5b compared with Fig. 5a. The majority of dark lines in the MQW CL image (Fig. 5a) of the sample without the pre-layer can be associated with misfit dislocations forming at the interfaces of the MQW structure generated by slip in the (0001) basal plane, since these dark lines do not appear in the CL intensity image of the GaN NBE emission (Fig. 5c). The more pronounced dark line on the right hand side of the Fig. 5c is attributed to a stacking fault as it appears at the same place in both the GaN NBE and MQW intensity images. In contrast to stacking faults, misfit dislocations do not penetrate through the entire layer. They are generally generated at interfaces when a threading dislocation bends into the plane of the interface. Hence, a high density is observed in the MQW intensity image, but not in the GaN NBE intensity image. Comparison of the MQW intensity images of the samples with and without the pre-layer shows that the sample with the pre-layer exhibits a higher density of dark lines. This indicates that the sample with the SLS pre-layer has a higher misfit dislocation density than the sample without a pre-layer. Additionally, the CL intensity image generated from the SLS peak (Fig. 5d) shows a high density of dark lines, implying that a large number of misfit dislocations were generated at the interfaces in the SLS pre-layer even before the growth of the MQW structure. The generation of misfit dislocations for the sample without a pre-layer is largely due to the InGaN/GaN MQWs as a result of the large lattice-mismatch between InGaN and GaN. However, the enhanced misfit dislocation observed on the sample with the SLS pre-layer is clearly due to the SLS pre-layer. The critical thicknesses for stress relaxation via the formation of misfit dislocations depends on growth orientation. Detailed studies confirm that the critical thickness of InGaN grown on (11–22) GaN is much thinner than for growth on *c-plane* GaN or (20–21) semi-polar GaN^26–30^. Therefore, although the InN content in the InGaN of the SLS pre-layer is as low as 3%, misfit dislocations are still generated. It therefore can be concluded that the insertion of the SLS indeed generates extra misfit dislocations.

Based on previous studies, point defects such as nitrogen vacancies or surface point defects generated during high temperature growth of GaN layer can be captured by a low-temperature grown InGaN pre-layer^8–10^. As a result, the performance of the semi-polar (11–22) LEDs with the SLS pre-layer can be improved due to a reduction in point defect density. However, the insertion of the SLS also introduces extra misfit dislocations acting as NRCs, thus leading to a reduction in optical performance. The consequence of the competition of these two mechanisms leads to an improvement in optical performance, but the improvement is not as significant as that observed for its *c-plane* counterparts.

## Conclusions

This paper has systematically investigated the influence of an InGaN/GaN SLS pre-layer inserted prior to the growth of the InGaN/GaN MQWs of a semi-polar (11–22) LED on patterned Si (113) on the optical performance. It was demonstrated that the semi-polar LED with the SLS pre-layer exhibits an improvement in both IQE and light output as expected, which is similar to its *c-plane* counterparts. However, unlike *c-plane* LEDs, the SLS pre-layer also introduces extra misfit dislocations. Consequently, the performance improvement is not as significant as that for its *c-plane* counterparts. For example, the IQE can be enhanced from 13% for the semi-polar LED without a pre-layer to 23% for the semi-polar LED with a SLS pre-layer (factor 1.8), while the enhancement in IQE for its *c-plane* counterpart can be up to a factor of 3.5.

## Methods

*Photoluminescence measurements *have been conducted as a function of temperature. The samples are held in a helium closed-circuit cryostat in a temperature range from 10 to 300 K. A 375 nm diode laser is used as an excitation source. The luminescence is dispersed by a 0.55 m monochromator and then detected by a Jobin Yvon CCD.

*Time resolved PL measurements* have been carried out as a function of temperature by using a time-correlated single photon counting (TCSPC) system, where a 375 nm pulsed diode laser with a pulse width of 83 ps and a pulse period of 50 ns is used as excitation source, and the emission is detected by a Hamamatsu hybrid photon counting PMT. The response time of the system is 150 ps. The samples are held in a helium closed-circuit cryostat.

*Device fabrication* was performed on both semi-polar green LED epi-wafers in a same batch for detailed comparison by means of using a standard photolithography technique. Lateral LED mesas with a typical size of 330 × 330 µm^2^ were formed using dry etching. A highly transparent Ni/Au layer and a Ti/Al/Ti/Au alloy have been deposited by a thermal evaporator and then annealed by rapid thermal annealing in order to form p-contact and n-contact, respectively. A Ti/Au alloy has been finally deposited as pad electrodes for both p-type and n-type contacts.

*Electroluminescence measurements* have been performed on the bare chip LEDs as a function of injection current at room temperature in a continuous current injection mode using a Keithley 2,400 source meter.

*Cathodoluminescence (CL) measurements* have been carried out at room temperature using a field emission gun scanning electron microscope (SEM). Samples are placed in the SEM chamber, and are tilted by 45°. The emission is collected by a Schwarzschild reflecting objective with its optical axis perpendicular to the direction of the electron beam, and then dispersed with a 1/8 m focal length spectrometer, and finally detected by a 1,600-channel electron multiplying charge-coupled device.
